# A Biochemical Analysis of LINC00298 RNA's and How It May Contribute to the Development of Alzheimer's Disease

**DOI:** 10.1002/alz70855_099861

**Published:** 2025-12-23

**Authors:** Rachel A Hellmann Whitaker

**Affiliations:** ^1^ University of Minnesota Medical School, Minneapolis, MN, USA

## Abstract

**Background:**

Long non‐coding RNAs (lncRNAs) are ubiquitous throughout the human system, yet many of their biological functions remain unknown. LINC00298 RNA, a long intergenic non‐coding RNA (lincRNA), has been shown to have preferential expression in the Central Nervous System (CNS) where it contributes to neuronal differentiation and development. Furthermore, previous research has indicated that LINC00298 RNA is known to be a genetic risk factor for the development of Alzheimer's disease. Herein, we biochemically characterize LINC00298 RNA and to elucidate its biological function within hippocampal neuronal cells, thereby providing a greater understanding of its role in Alzheimer's Disease pathogenesis.

**Method:**

LINC00298 RNA was *in vitro* transcribed and then subjected to structural analysis using Circular Dichroism, and UV‐Vis Spectroscopy. Additionally, affinity column chromatography was used to capture LINC00298 RNA's protein binding partners from hippocampal neuronal cells, which were then identified using Liquid Chromatography (LC) and Mass Spectrometry (MS) (LC/MS).

**Result:**

LINC00298 RNA is comprised of stem‐loop secondary structural elements, with a cylindrical tertiary structure that has highly dynamic regions, which result in high positional entropy. LC/MS identified 24 proteins within the interactome of LINC00298 RNA.

**Conclusion:**

Through analysis of LINC00298 RNA's 24 protein binding partners, it was determined that LINC00298 RNA may play significant roles in neuronal development, proliferation, and cellular organization. Furthermore, analysis of LINC00298 RNA's interactome indicated that LINC00298 RNA is capable of intracellular motility with dual localization in the nucleus and the cytosol. This biochemical characterization of LINC00298 RNA has shed light on its role in Alzheimer's Disease pathogenesis.

